# Privileges among the privileged: the effect of starting conditions at age 16 on occupational success from age 16 to 66 in an educationally privileged group

**DOI:** 10.3389/fsoc.2025.1568400

**Published:** 2025-07-30

**Authors:** Klaus Birkelbach, Hermann Dülmer, Heiner Meulemann

**Affiliations:** ^1^Faculty of Educational Sciences, University of Duisburg-Essen, Essen, Germany; ^2^Faculty of Management, Economics and Social Sciences, University of Cologne, Cologne, Germany

**Keywords:** occupational prestige and income, age effects, life course age 16 to 66, panel analysis, high school students, Germany 1969–2020

## Abstract

**Introduction:**

Do starting conditions in an educationally selected youth still affect occupational success in adult life? Educational selection depends on starting conditions for the career beyond the school which are *given* or *controllable* – gender, intelligence, and social origin vs. grades, aspirations, and life plans. In a group already selected, it intensifies competition and challenges motivations to succeed more strongly such that new privileges can arise among the already privileged. This is examined longitudinally from age 16 to 30, 43, 56, and 66. Two hypotheses will be tested cross-sectionally at each age: (1) *Effectiveness*: All starting conditions should increase occupational success, (2) *Control force*: Given starting conditions have less impact than controllable ones. And two hypotheses will be tested longitudinally from age 30 to 66: (3) *Tapering off*: All starting conditions lose impact. (4) *Control persistency*: Given conditions lose more impact than controllable ones.

**Methods:**

Data are from the *Cologne High School Panel* (CHISP). It starts off with 3240 German Gymnasium (the highest layer of the three German high school forms) students at age 16 in 1969. They have been re-interviewed at age 30, 43, 56, and 66 when 1013 respondents remain. The *occupational career success from 16 to 66* is measured as occupational prestige and hourly net income, corrected for inflation.

**Results:**

The results show that prestige is higher for men than women; it increases with social origin, and aspirations to a strong degree at age 30, to a lesser degree at age 43, to a still lesser degree at age 56, and not at all at age 66; however, the impact of the earlier success increases strongly and continuously. In brief, the past fades away and the careers consolidate. But given conditions do not have weaker impacts than controllable ones and do not lose their impact more strongly. Thus, (1) the *effectiveness hypothesis* is confirmed for most starting conditions, but (2) the *control force hypothesis* is not; and (3) the *tapering off hypothesis* is, but (4) the *control persistence hypothesis* is not. Given starting conditions have no less power over occupational success than controllable ones. Privileges resonate indiscriminately and decreasingly in life histories.

**Discussion:**

Income at age 30, 43, 56, and 66 does not increase continuously with any starting condition, but decreases with intelligence at age 30 and increases with male gender and having a life goal at age 43. And the impact of the earlier successes increases strongly and continuously. In brief, the past throws no shadow and the careers consolidate. The (1 and 2) *effectiveness and the control force hypothesis* are disconfirmed. Given the irregular impacts of starting conditions, (3 and 4) the *fading off and the control persistency hypothesis* cannot be meaningfully examined. Privileges do not continue to favour the privileged and occupational careers stabilize autonomously.

## 1 Research question

Educational selection separates more successful students from less successful ones. The positively selected students should outperform those left behind, but they should also be more homogeneous. Among those privileged by educational selection, the mean of achievement, school grades, and test intelligence should be higher than in the student population at large, with a lower standard deviation. Similarly, social resources that partly determine achievement should also be greater in the privileged group than in the general student population. However, this does not mean that achievement and social origin no longer filter the student population; they continue to differentiate careers and may further privilege the already advantaged by creating “cumulative advantages” (DiPrete and Eirich, 2006, p. 286; Schafer et al., 2011, pp. 1056–7). “Existing social inequalities are deepened further because it is already privileged social groups that profit most from atypical paths to higher secondary education” (Buchholz et al., 2016, p. 90). These factors have consequences for occupational careers and societal inequality at large (Triventi et al., 2016, pp. 4–6, 12–13).

In education and elsewhere, advancement intensifies competition. Thus, the *intensification of competition* is the mechanism that creates career differences among already selected students. As the educational and later on the occupational career progresses, resources for career success are increasingly mobilized, whether intentionally or not. In particular, *starting conditions* continue to demonstrate their impact and importance; they remain prominent in students’ minds and gain power over their lives. The following research investigates how four of these starting conditions—achievement, motivation, social origin, and personal qualities—affect *occupational career success* from youth to retirement.

First, achievement is the primary and legitimate criterion of occupational success. According to this criterion, schools and firms assign certificates and rewards, and students and workers plan their careers.

Second, motivation is a legitimate but secondary criterion of occupational success. It may lead to success, but cannot compensate for a lack of achievement. Increased competition may reveal previously hidden ambitions among students, which can serve as incentives for independent work and learning, irrespective of school grades. In particular, *aspirations* can reveal their motivational force and manifest in subsequent careers (Schoon and Lyons-Amos, 2016, p. 13).

Third, social origin is not a legitimate criterion, but a powerful determinant of occupational success. It influences achievement and fosters motivation, but cannot substitute for them. Increased competition may mobilize support from students’ families that was previously taken for granted. Students may leverage their families’ financial resources and social connections to gain competitive advantages through home education, travel, and cultural participation. Thus, *social origin* can be transformed into new privileges.

Fourth, similar to social origin, personal qualities—such as gender and ability—are not legitimate criteria but powerful determinants of occupational success. Men and women may respond differently to career challenges and may be treated differently throughout their careers. Ability is a prerequisite for achievement. Thus, while gender should not be a criterion for success and ability cannot be, both may still confer new privileges.

The impact of starting conditions on occupational success from youth to retirement is investigated in a privileged starting: 10^th^-grade students of the German *Gymnasium.* This is the highest tier among the three German high school forms—above the *Realschule* and *Hauptschule.* It is the only type that provides direct access to universities via the *Abitur,* the high school graduation after grade 13 (Schneider, 2008, p. 512). In 1965, when our sample was in grade 6, it was attended by only 19% of 8^th^-grade students, the *Realschule* by 15%, and the *Hauptschule* by 66% (BMBF, 2023, p. 34). By 1969, it held a near monopoly of about 92% in providing eligibility for tertiary education (Schindler and Bittmann, 2023, p. 208).^1^

Occupational success is measured in terms of prestige and income. The former captures the social recognition of occupations, while the latter reflects their economic productivity. Within a given occupation, income serves as a reward for abilities and motivation. Moreover, different income classes encompass occupations from various economic sectors with distinct productivity levels and career management rules. Therefore, both need not be “consistent,” that is, they do not necessarily place people at the same relative level.

In summary, the research question explores to what extent achievement, aspirations, social origin, and personal qualities consistently affect occupational prestige and income from youth to retirement in a group of students already selected based on these criteria.

## 2 Selections after selection according to achievement, aspiration, social origin, gender, and ability: a review of literature

As our sample is drawn from the highest branch of the German tripartite secondary school system, the *Gymnasium,* the first part of the following literature review is limited to studies that have examined the effect of starting conditions on *already privileged life careers in the German educational system*. Additionally, as aspirations have been researched less often than achievement and social origin in Germany, the second part of the review focuses on studies concerning their long-term effects in *unselected student samples in other countries*.

### 2.1 Starting conditions: effects on careers in the German socially selected secondary school branches

The few studies that have examined the effects of one or more of the three starting conditions on *already privileged life careers in Germany* (Wohlkinger and Ditton, 2023, p. 183) are presented as follows. The dependent variable, life career, is organized roughly according to the sequence of transitions after entering the secondary school branches. The independent variables are reported according to their meritocratic legitimacy in determining school outcomes: achievement precedes motivation and social origin. Consequently, studies are divided into three groups.

The first group of studies follows *secondary school careers* longitudinally and examines the effects of *achievement* and *social origin*. Three of these studies investigate upward and downward movement between the three branches of the German secondary school system.

First, in a panel study of 3^rd^-grade pupils until the 9^th^-grade regarding their school mobility between the 5^th^ and 9^th^ grades, *upward moves* from *Hauptschule* and *Realschule* to *Gymnasium* increased with grade point averages and parental education, while *downward moves* from *Gymnasium* to *Realschule* or *Hauptschule* decreased with grade point averages and parental education (Buchholz et al., 2016, p. 87).

Second, the *National Educational Panel (NEPS),* a retrospective survey of 9^th^-grade students conducted in 2010 regarding their secondary school career from 5^th^ to 9^th^ grade, revealed that *downward moves* from *Gymnasium* to *Realschule or Hauptschule* between grades 5 and 9, and between 2008 and 2013, decreased with competence test scores in reading, mathematics, and science, as well as with parental education, and were more frequent among boys than girls (Wohlkinger and Ditton, 2023, p. 197). Both studies demonstrate strong effects of achievement—tests or grades—and the persistence of social origin effects, even when controlling for achievement.

Third, a similar retrospective NEPS survey of 9th-grade students in 2010 on their secondary school careers from 5^th^ to 9^th^ grade showed that re-entering secondary school at the *Gymnasium* level after graduating from *Hauptschule* or *Realschule* increased with grade point averages and was almost equally influenced by parental education (Buchholz et al., 2016, p. 88).

These three studies indicate that movements within the three branches of the German secondary school system, while governed by achievement, remain influenced by social origin.

A fourth study compares competencies of students from grades 7 to 9 across different tracks leading to the *Abitur*. Students in the more prestigious humanistic and scientific *Gymnasium* tracks exhibited an *advantage in reading and mathematical skills* compared to students in alternative—mostly vocational—tracks. This advantage diminished but remained significant when controlling for social origin, migration background, and competencies prior to entering secondary school (Schindler and Bittmann, 2023, pp. 219–20). Thus, even in higher and already selected grades, students in the *Gymnasium* maintain competence advantages over alternative tracks to the *Abitur.*

In summary, even in already selected branches after grade 9, educational success depends, as it should, on achievement, but also on social origin.

The second group of studies provides longitudinal information on the effects of *aspirations* on Gymnasium careers. It comprises three studies, two of which again use the *NEPS.*

First, *educational aspirations* expressed in higher levels of certification and *occupational aspirations* expressed in higher prestige levels were examined among 9^th^-grade students at age 15 in 2010 and re-interviewed at age 20 in 2015. Both aspirations were higher among students who had attained an intermediate certificate upon leaving school in grade 9 than among those who had attained a low certificate (Holtmann et al., 2023, p. 276, 281). As the respective percentages were computed based on the dependent variable, this suggests an indirect effect of aspirations on school careers. Until 2015, furthermore, 70% of those with a low certificate remained in that category, while 34% of those with an intermediate certificate achieved what they had considered realistic in 9th grade (Holtmann et al., 2023, p. 281–85). Both results illustrate the motivational power of educational aspirations, although indirectly.

Second, *aspirations for the Abitur* persisted between grades 5 and 9 in the *Gymnasium* but dropped by 25 percentage points in alternative tracks. This difference decreased but remained significant when controlling for social origin, migration background, and competencies (Schindler and Bittmann, 2023). Although this result again refers to the difference between the *Gymnasium* and alternative tracks to the *Abitur*, it highlights the aspiration advantages of the former, which were gained and maintained during secondary school.

In a third study, “*Arbeit und Lernen im Wandel*” (ANWA, “Work and Learning in Change”), *aspirations for any occupation (Berufswunsch)*, regardless of its prestige level (Pollak et al., 2011, p. 87, 92), had no impact on the time span between graduating from secondary school and entering occupational education, including university. Similarly, the already-selected grade 9 students from the study mentioned above (Holtmann et al., 2023, p. 281) reported the same frequencies of desired occupations at each level of prior certification.

In sum, aspirations across a range of status levels and degrees, that is, educationally defined aspirations, appear to affect school careers—but this could only be demonstrated indirectly. Directly measured aspirations for a specific occupation, that is, self-defined aspirations, have no effect on school careers. Aspirations draw motivational power primarily from the notion of a scale of advancement rather than from a preference for a personal goal. In none of the above studies has their effect on careers been shown directly and convincingly.

The third group of studies only analyzes the effects of *social origin* and often gender on secondary school careers, without controlling for achievement and aspirations. It comprises five longitudinal studies.

First, the higher the prior probability of access to high school, the less likely it was that students from the *German Socio-economic Panel (G-SOEP)* would drop out during the *Gymnasium* from grades 5 to 9 between 1984 and 2006—as was also the case during *Hauptschule* and *Realschule*. It was less probable in the salariat than in the working class; it was less likely the higher the parents’ education; and it was lower for girls than for boys. Finally, survival in high school decreased continuously over the years spent there (Schneider, 2008, p. 516, 521–3).

Second, among students aged 17 to 19 from the *Mikrozensus* of the German Statistical Office in 2008, attending the *Oberstufe* (last three grades) of the *Gymnasium* instead of an occupational school, or leaving the *Oberstufe*, increased significantly with the education of both the father and mother and was higher for girls than for boys (Pollak et al., 2011, pp. 76–8). Thus far, *social origin* and *female gender* still determine social selection after the initial selection into the Gymnasium.

Third, in the NEPS, *re-entry* after leaving *higher education* and *final dropout* was assessed in a series of retrospective surveys from 2010 onwards among university or *Fachhochschule* students from birth cohorts 1944 to 1984. Re-entry after leaving was more probable among students with parents who had higher education—and final dropout was less likely; furthermore, re-entry was less probable among girls than boys (Tieben, 2023, p. 229, 236, 238).

Two further studies analyze the transition times between career steps in the ANWA study of 10,000 students from birth cohorts 1956 to 1988 conducted in 2007–8.

Fourth, the *time between graduating from secondary school and entering an occupational training including university* is shorter and more often performed after the *Abitur* than after graduation from the *Realschule* and again after the *Hauptschule.* Seemingly, being in the 10^th^ grade of the Gymnasium—which leads to the *Realschul* graduation as well as to the *Abitur*—facilitates the entry into occupational education; and shorter transition times constitute an advantage of higher graduation levels. However, this transition time was not consistently affected by the mother’s and father’s education, and not at all by the occupational aspiration (*Berufswunsch*) (Pollak et al., 2011, p. 87, 92).

Fifth, the *time between occupational training and employment* is shorter and more often successfully concluded after obtaining a university diploma than after other forms of occupational education; the 10th grade of the Gymnasium—which predominantly leads to an *Abitur* and university studies—also facilitates entry into employment. Moreover, for women, this transition time is independent of prior educational achievement and of the mother’s and father’s education. For men, however, it decreases from graduation in *Hauptschule* to *Realschule* and then to *Abitur*, and it increases with a university diploma (Pollak et al., 2011, p. 96, 99). Seemingly, transition times are not inherently advantageous, but dictated by the specific requirements of their targets.

If one overlooks the dependent and independent variables of the presented research on privileged careers in Germany, some generalizations can be ventured.

As for the dependent variables, school career decisions—leaving and re-entering, switching and dropping out—are predominantly examined. Decisions on entering employment and occupational advancement are less frequently studied, and success in occupational life is almost never addressed. In brief, the further the career progresses in the already selected group of secondary school students, the less it is examined. The effects of starting conditions on many later career steps remain to be detected.

As for the independent variables, the first legitimate criterion—achievement—retains its positive effect even when controlling for social origin. Grades as well as test scores consistently have positive effects. Although a legitimate criterion as well, aspirations are the least researched. Educationally defined aspirations had, if adequately measured, an effect on careers in only one study, while self-defined aspirations had no effect.^2^ Social origin is often still examined in isolation, probably because it has already been effective in earlier selection and has lost power. Across studies, its effect remains ambiguous.

### 2.2 Aspirations: effects on careers in socially unselected student samples of other countries

In contrast to the German studies cited above, *educational aspirations* did affect later educational and occupational careers in a British panel of age cohorts born between 1980 and 1989. They were assessed at age 14 with the question: “Do you want to leave school when you are 16, or do you plan to carry on in education, for instance in the sixth form or a college?” Responses were coded as “stay in education,” “do not know,” and “leave.” As a dependent career variable, transitions between ages 17 and 23 from 1996 to 2007 were analyzed and summarized into four clusters reflecting a hierarchy of future career prospects: “early work orientation,” “employment after some education,” “exclusion from employment,” and “extended education.” In a multinomial logistic regression of the highest cluster - “extended education” - against the three lower career clusters, the aspiration to “stay in education” had a positive effect compared to “do not know” and “leave,” even when controlling for parental social class, parental education, and gender. Social class and parental education consistently had positive effects, while gender had none. When the regression coefficients were transformed into probabilities of cluster membership from age 17 to 23, they decreased by 9 percentage points in the “early work” cluster, developed inconsistently in the two middle clusters, and increased by 25 percentage points in the “extended education” cluster (Schoon and Lyons-Amos, 2016, pp. 15–8). Thus, properly measured and analyzed aspirations can significantly affect later life careers.

Educational aspirations are an early expression of *motivations.* In a sample restricted to men aged 21 to 29 from the 1968 to 1971 waves of the *Panel Study of Income Dynamics* (PSID), it was measured by agreement with the question “Do you plan for the future?” which was averaged over the five waves. It correlated at approximately r = 0.20 with the average hourly log wage from 1988 to 1992. Together with positive responses to questions about whether one thinks life will work as wished and whether one usually finishes things one has started, it was combined into an index of *personal control*. This index had a significant positive effect on *wages* from 1988 to 1992 when controlling for years of education, verbal intelligence, age, father’s occupation, region of origin, and health. The effect was reduced but remained significantly positive when additionally controlling for the initial 1968–1972 wage. The effect did not decrease when income was measured 15 years earlier and when motivation was measured in a cohort 10 years older (Dunifon and Duncan, 1998, p. 38, 41–3). Thus, planning has a persistent positive rather than a diminishing effect on success in later life.

In summary, studies from other countries provide more evidence on the impact of aspiration and motivation on career success than the German studies. It is possible that aspirations have already been expressed in the choices of branches of German secondary education, leading to an attenuated impact.

## 3 Design of study

### 3.1 Data and dependent variables

The data are from the Cologne High School panel (CHiSP), which began in 1969 with 3,240 10^th^-grade students from 121 classes in 68 high schools (*Gymnasium) in the federal state of North Rhine Westphalia*, at a median age of 16 (Meulemann, 1979, p. 191). In this sample, called the primary survey or PS16, the starting conditions were surveyed. In four follow-up surveys at ages 30, 43, 56, and 66, called FU30, FU43, FU56, and FU66, occupational success has been ascertained. The analysis sample consists of 1,013 respondents remaining in FU66.^3^

The dependent variable, occupational success, comprises occupational prestige (MPS) and net household income (HINC).

#### 3.1.1 Prestige and income: conceptual differences

Prestige and income differ in two respects.

*First, gravity of prestige—volatility of income.* Prestige is a sociological scale capturing the social appreciation of occupations. It remains unaffected by many moves within an occupation. Occupations are internally differentiated according to seniority and salary class, as in the public sector, for example. Moreover, occupations are often chosen early in life, leading to limited revisions. For the former 10^th^-grade *Gymnasium* students of the CHiSP, the range of available occupations is elevated, such that positional moves are often not reflected in prestige differences. This causes prestige to increase significantly between ages 30 and 43 when occupations are entered but only slightly later when careers stabilize. Once a first prestige is attained, the range of additional moves is rather narrow. Thus, starting conditions can have a considerable impact at the beginning of a career, but not later on. Income, on the other hand, is a social scale that serves as a seismograph of the vicissitudes of the occupational career. Booms and busts of a firm or the economy at large can trigger upward and downward moves in the same occupational position. As a result, the effects of starting conditions may be diminished.

*Second, codification of prestige—negotiability of income*. Prestige is codified in society as a societal reputation assessment before being scientifically reconstructed as a scale; income is defined and negotiated using a social unit, money, whose lower and upper limits, zero and infinity, are known to everyone. There can be no dispute about prestige between employer and employee, but there is often much discussion about income. People tend to take prestige for granted but are eager to improve their income. Thus, prestige is less affected by personal endeavors than income. Achievements during occupational life interfere less with prestige than with income, allowing starting conditions to have a greater influence on prestige than on income development.

Because prestige is sociologically captured on a scale, its lower and upper limits are known only to its constructors. If an occupation enjoys extreme social recognition, its prestige score may not exceed that of a somewhat less appreciated occupation to a remarkable degree such that moves at the top of social appreciation may remain undetected on the sociological scale. A similar fuzziness exists at the lower end of social appreciation. In both cases, small moves within and between occupations may not translate into score differences. Thus, effects of starting conditions can appear early and survive diminished later on.

Since the base of prestige, appreciation in society at large, is remote from everyday life, most people are willing to disclose their occupation. Income, however, is “conspicuous” in consumption decisions. When stated to the interviewer, it reveals occupational success, leading to shame or anxiety that may cause respondents to over- or underreport it or refuse to respond. In summary, prestige is likely measured more reliably than income. Effects of starting conditions may be obscured by the noise in the measurement of income.

#### 3.1.2 Prestige and income: measurement

In each follow-up, the occupational career has been assessed retrospectively using the same inventory. Respondents had to divide their career into episodes with monthly starting and ending points. For each episode, the position, monthly income, and working hours were recorded; the position was measured by Wegener’s (1988, pp. 168–174; 1992, pp. 253–254, 269–271) *Magnitude Prestige Scale, MPS*.^4^ The income as *hourly net income, corrected for inflation (HINC)*. As each wave covered several episodes, there were three ways to choose a value for the wave from the episodes. The *current* value at the waves referred to the exact time of the interview but had frequent missing values, and the *highest* values varied too much between episodes; therefore, the *last* value was chosen.

Within waves, MPS and HINC correlate rather weakly: r = 0.218 at age 30, r = 0.104 at age 43, r = 0.216 at age 56, and r = 0.214 at age 66. There is considerable room for inconsistency at each age and for changes over the life course. However, MPS correlates much more strongly across ages than HINC: r = 0.720, 0.879, and 0.970, in contrast to r = 0.146, 0.557, and 0.857. Prestige seems to be the theme of occupational development, while hourly income shows more variation. This suggests that starting conditions have a stronger impact on the more stable occupational dimension, that is, more on MPS than on HINC.

Across all waves, respondents reported up to 22 occupational episodes with individual starting and ending points. To cover the entire range between ages 16 and 66 on the same monthly scale for every respondent, MPS and HINC values between starting and ending points have been interpolated linearly, resulting in *monthly values between ages 16 and 66* resulted.

### 3.2 Prestige and income over the life course

**Figure 1:**
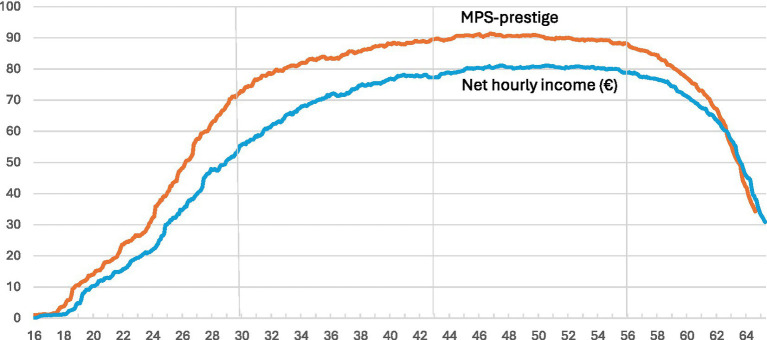
Responses to MPS prestige and net hourly income in € (HINC) among the full- and part-time employed 1,013 participants at each month between age 16 and 64, in percent.

**Figure 2:**
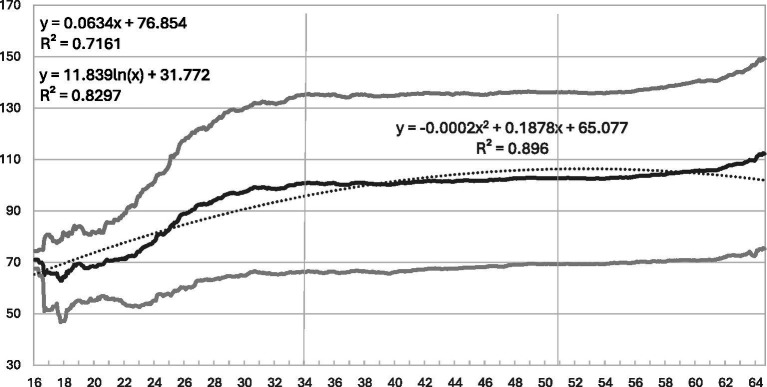
MPS at each month from age 16 to 64 among 1,013 respondents: means, standard deviations above and below, and fitted quadratic curve.

**Figure 3:**
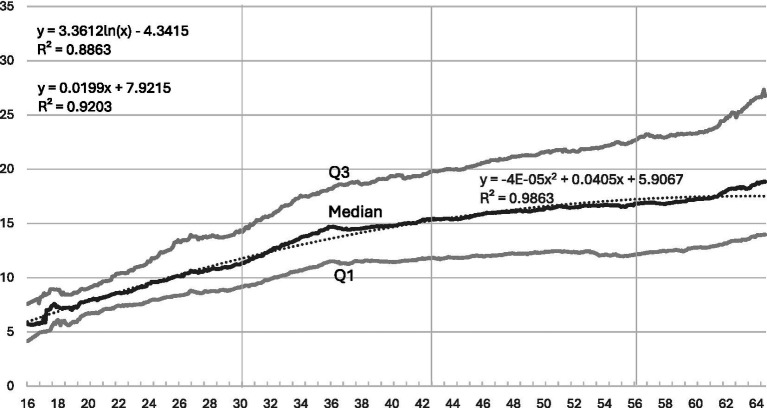
HINC at each month from age 16 to 64 among 1,013 respondents: median, first and third quartile, and fitted quadratic curve.

The monthly response rates of the 1,013 respondents in each month between ages 16 and 64, which allow for the coding of MPS and the computation of HINC, are presented in Figure 1. It spans from PS16 to FU66. The in-between follow-ups FU30, FU43, and FU56 are marked by vertical lines; the same applies to Figures 2, 3.

MPS responses increase strongly and continuously from age 16 to 30, remain around 90% during midlife, and decrease significantly after age 55. This primarily represents the life cycle employment curve, but partly it is also due to missing values. The HINC responses increase continuously from age 16 onwards, remain constant in midlife, and decrease after age 55. The HINC response curve for income mirrors the one for MPS, yet it remains consistently lower. The distance increases from zero at age 16 to roughly 10 percentage points during midlife and decreases to 4 percentage points at age 64. Respondents frequently refuse to disclose their income even when they reveal their occupation.

Prestige and income can be expected to follow different developmental patterns. Prestige is attained with the choice of an occupation relatively early in the career, increases only slowly, and has an upper limit. Income is relatively low in early career phases, increases, and has no upper limit. Thus, increments should be expected for both prestige and income, but weaker and more strongly decelerating ones for prestige.

The means of MPS in each month between ages 16 and 66 are presented in the solid line of Figure 2. They drop from 79 to 64 between ages 16 and 18, increase to 95 by age 30, remain constant until age 60, and then increase again to 110 by age 64. Thus, apart from the early and late ages when many have not yet entered or have already left occupational life, these results confirm our expectation: Prestige is gained until age 30 and maintained thereafter. Because MPS varies within constant lower and upper limits of 20.0 and 186.8, the dotted lines representing one standard deviation below and above the mean diverge from the mean only up to age 30 and then accompany it at constant distances.

The gravity of occupational prestige over the life course can be discerned from the horizontal lines in Figure 2. The mean MPS increases more gradually, from 95.3 to 100.7, 102.2, and 103.9; and its standard deviation remains almost constant at 32.4, 34.0, 33.7, and 33.8. The total increase in MPS between ages 30 and 66 of roughly 8 points amounts to only a quarter of the standard deviation, which averages around 33 points. Finally, the correlations between the MPS scores at earlier and later ages are relatively high, increasing from r = 0.714 for ages 43 and 30, to r = 0.880 for ages 56 and 43, and r = 0.912 for ages 66 and 56.

A linear, quadratic, and logarithmic regression of the MPS means for each month were computed. Of these, the quadratic and logarithmic equations should perform better than the linear one because they capture the early increase and the later constancy. According to the R^2^ values, the quadratic equation performs best. MPS increases from 65 at age 16 by 0.19 units per month and decreases by 0.0002 per month squared. Its fitted values, represented by the narrowly dotted curve in Figure 2, show a linear increase that bends slightly at both early and late ages. Were it not for the slight increase in the empirical values after age 60, the predicted development of MPS could be interpreted as a case of “diminishing marginal utility” (Samuelson and Nordhaus, 1998, pp. 81–2) over time.

The median^5^ of HINC in each month between ages 16 and 64 is presented in Figure 3, again shown as a solid line. It increases significantly from 6 to 15 € between ages 16 and 36, and more modestly to 18 € at age 64. This confirms our expectation: HINC improves over the entire life course, but at a decreasing rate. Its median, as well as its first and third quartiles, increases from ages 16 to 66—just as they do in a shorter panel from ages 20 to 35 (Hitlin and Johnson, 2015, p. 1449). Because income has a lower but no upper bound, the curves of the first and third quartiles diverge from the median throughout the life course. One can always improve one’s salary within a constant position.

The volatility of HINC over the life course can be inferred from the horizontal lines in Figure 3. The means and standard deviations of HINC increase almost continuously: from 12.05 to 16.62, 19.87, and 22.19 €, and from 6.81 to 18.01, 24.46, and 28.03 €. The total increase in HINC income between ages 30 and 66, approximately 10 €, is larger than the standard deviation of less than 7 € at age 30, when the occupational career begins. Moreover, the standard deviation grows threefold by age 64, when careers have diverged asunder. Finally, the correlations between HINC at earlier and later ages are only moderate, increasing from r = 0.151 to r = 0.573 and r = 0.857. In comparison with the corresponding correlations of MPS, the first two are considerably lower, while the last one is somewhat lower. The gravity of occupational prestige contrasts with the volatility of income up to age 56. After that, people begin to retire, and the last income value may refer to the retirement period, moving closer to the last income value at age 56, resulting in greater income stability as both dimensions stabilize nearly equally.

Again, a linear, quadratic, and logarithmic regression were computed. Again, the quadratic and logarithmic equations, which capture the decreasing rate of increase, should perform better than the linear one. Indeed, the quadratic equation performs best and is again presented as a dotted curve. The median of HINC increases from 6 € at age 16 by 0.04 € per month and decreases by 0.00002 € per month squared. The fitted curve is predominantly linear with only slight downward bends at the ends. It closely reproduces the empirical values throughout the entire life course.

Summarizing the monthly developments, the different qualities of prestige and income are revealed in three forms. *First*, the developmental form: Prestige increases up to age 30 and remains constant thereafter, while income increases throughout the entire life course. *Second*, the regression coefficients: The quadratic and linear coefficients of age are larger for prestige than for income, reflecting the different developmental forms. Prestige increases more sharply at the beginning and declines more steeply at the end of the occupational career. *Third,* the explained variance: It is smaller for prestige than for income. Prestige increases depend more strongly on factors beyond its conceptual base appreciation, such as personal networks and workplace opportunities; income increases more strongly the higher its already achieved level.

### 3.3 Hypotheses and independent variables

Rather than focusing on the monthly developments, the hypotheses are tested for the development between the waves, indicated by the first and last month and the vertical lines in Figures 1–3. The question of whether starting conditions differentiate careers is addressed from two longitudinal perspectives.

*During the career take-off* from youth to early adulthood or from PS16 to FU30, starting conditions are most powerful. Although high school and university attendance may delay the career start relative to the general student population, most former high school students are employed in some occupation by age 30. The question then becomes which conditions are the most effective. The answer requires comparing standardized coefficients from OLS regressions of MPS and HINC at age 30 based on starting conditions at age 16.

*During the life-long career* from youth to retirement or from PS16 to FU30, FU43, FU56, and FU66, the question is which effective starting conditions maintain their impact. The answer necessitates a hierarchical linear multilevel regression (HLM) with MPS and HINC at the four follow-ups on level 1 and the starting conditions of PS16 on level 2. As age generally favors occupational progress beyond specific starting conditions, it must be introduced into the HLM before the starting conditions. However, to test how much of the age effect on occupational success is accounted for by starting conditions or to determine the effect of starting conditions net of age, both must be introduced simultaneously.

The starting conditions surveyed in PS16 are classified together with hypotheses and variable names in Table 1. The impact of starting conditions on the career take-off will be justified first, followed by their impact on the life-long career.

**Table 1:** Classification of starting conditions at age 16, hypotheses about their effects on occupational success from age 16 to 66, and variables available in the CHiSP at age 16.

Control dimensions			Hypotheses				Variables in PS16
			Career take-off		Life-long career		
			E	CF	T	CP	Name
Given	Personal	Gender	+	0	−	0	MALE
		Intelligence	+	0	−	0	IST
	Social	Social origin	+	0	−	0	FAPREST
Controllable	Achievement	Grades	+	1	−	1	AVGRADE
	Motivation	Life planning: aspiration	+	1	−	1	ABIOPEN, ABISTUD
		Life planning: life goal	+	1	−	1	LIFEGOAL

E Effectiveness, + positive effect. CF Control force 0 smaller, 1 bigger effectiveness. Tested in OLS regressions. T Tapering off, − decrease. CP Control persistence 0 weak (stronger decrease), 1 strong (weaker decrease). Tested in HLM regressions.

#### 3.3.1 Starting conditions during career take-off: effectiveness hypothesis and control force hypothesis

During the career take-off, starting conditions can be categorized into two types. Some are inherent to the person, such as gender, personality, intelligence, or attractiveness (Rosar et al., 2023). Others are achieved through goal-directed efforts. In either case, they contribute to attaining success. Thus, the *effectiveness hypothesis* (H1) states that starting conditions of the educational career positively affect the occupational career.

In the pursuit of occupational success, starting conditions vary in their power and legitimacy. Similar to conditions of action in general (Parsons and Shils, 1951, pp. 76–91), they can be divided into ascribed and achieved. All of them initially operate unnoticed; as a rule, students take their gender and social origin for granted and may be unaware of their test intelligence. However, some can be actively focused upon by the students. Thus, starting conditions differ in the strength of motivation they elicit (Blossfeld et al., 2023, pp. 7–8). If they are *given*—ascribed—to the person, their motivational power is low; if they are *controllable*—achieved—by the person, it is high. Given and controllable starting conditions have been primarily analyzed in non-selective samples according to the “Wisconsin Model of Socioeconomic Achievement” (Alexander et al., 1975, p. 325; Jencks et al., 1983, pp. 3–10). Most concepts of this model—except for the support of “significant others”—are also surveyed in this study. However, it specifically aims to test whether given and controllable starting conditions affect the occupational career differently, extending the dependent variable from occupational attainment between ages 25 and 30 to the occupational career from ages 16 to 66.

Given conditions are naturally distributed within a population, allowing their effects to be evaluated from the perspective of equality. They require some effort to manipulate and may not even be known to the bearer. They encompass inherited personal qualities—such as character and capabilities—that follow their own laws rather than the will of their bearer and are costly and risky to invest in.

Controllable conditions are not naturally distributed in a population, making their effects difficult to evaluate from the perspective of equality. They are largely subject to the willpower of their bearer. They include attainments, such as grades and knowledge, as well as motivations like resoluteness, diligence, perseverance, and aspirations. They manifest in constructed social facts: Students achieve results in the form of grades and express aspirations related to educational certifications and beyond.^6^ They are shaped by family and school during youth and pave the way for occupational success later on. They are instrumental for goal attainment and can be intentionally invested in. Thus, the general positive effect of starting conditions is differentiated according to the *control force hypothesis* (H2): Given starting conditions during youth increase occupational careers in adult life less strongly than controllable ones; in Table 1, weaker effects are marked with a 0, stronger ones with a 1.

The control force hypothesis (H2) can be more specifically justified for a pair of given and controllable starting conditions that are components of school performance, namely intelligence and grades. Intelligence is largely inherited and measured by test results that are mostly unknown to their bearers; it is a given condition of school performance. Grades depend largely on effort and are to some extent within the control of their bearers. They serve as focal points for life planning, along with the similarly controllable conditions of school aspirations and life plans. Moreover, as documents, they signal information to employers. Thus, the differential effects of control become particularly evident in two components of school performance: Test intelligence affects occupational careers less strongly than grades.

#### 3.3.2 Starting conditions during the life-long career: advancement hypothesis, tapering off hypothesis and preponderance hypothesis

During the life-long career, the career take-off continues, and the *effectiveness* (H1) and *control force* (H2) hypotheses remain under examination. However, time may diminish or even re-establish the effects of starting conditions. The question is when and which effects manifest. Therefore, hypotheses on three effects are proposed: of age as such, of age on starting conditions in general, and of age on given and controllable starting conditions.

Age comes with the accumulation of experiences, the gain of qualifications, and the acknowledgment of seniority by peers and employers. Therefore, it should increase occupational success. However, as experiences, qualifications, and seniority have their upper limits, their impact on occupational success should decelerate. The linear increase should be replaced by a quadratic decrease. Thus, the *advancement hypothesis* (H3) results: Age has a positive and decelerating effect on occupational success.

Starting conditions are a capital to be best utilized when available, in our case at age 16. However, their value may decrease with age. Additionally, with age, new conditions—stimulating achievements, mobilizing models, demanding relations, and generally encouraging experiences—emerge as competing capitals that may diminish the value of starting conditions. Some starting conditions, particularly social origin, may even favor a career anew in later stages of life. Furthermore, poor starting conditions that hinder a career might improve over time: Time heals many wounds. Yet, overall, the returns on starting conditions decay in the long run. Thus, the *tapering off hypothesis* (H4) results: The effects of starting conditions decrease with age.

Starting conditions are a safe and exhaustible capital. Yet given starting conditions are safer than controllable ones while controllable ones are more exhaustible. Given starting conditions remain the same throughout a career, while controllable ones can be used-up and re-loaded, they may affect occupational success differently. During youth, given starting conditions guide life plans. Gender norms influence choices of educational and occupational paths, intelligence opens learning potentials, and social origin provides models. However, after entering adult life, the guiding power of gender norms and intelligence, along with the resource of social origins, is largely exhausted (Schneider, 2008, pp. 521–26). Similar arguments apply to controllable starting conditions. In contrast to given conditions, controllable ones represent personal capabilities. Grades reflect not only intelligence but also virtues such as diligence, perseverance, and self-discipline; aspirations generalize from education to life; having life goals indicates self-assertiveness. As youth grow older and are challenged to plan and lead their lives independently, these capacities become increasingly important. Thus, the *control persistence hypothesis* (H5) results: Given starting conditions lose their impact on occupational career success more significantly than controllable ones; stronger decreases are designated by 0, and weaker ones by 1.

In summary, given starting conditions are assumed to have weaker and less persistent effects than controllable ones. Endowment should matter less than attainment, and nature less than merit.

#### 3.3.3 Independent variables

The starting conditions at age 16 are captured by the following variables: Gender: MALE = 1 (52.4%), female = 0. Intelligence was measured by four subtests of Amthauer’s Intelligence Structure Test IST (Meulemann, 1979, p. 194), which were calibrated^7^ to a mean of 100 and a standard deviation of 10, resulting in a mean of 112 and a standard deviation of 11.3 for 983 valid values in the educationally privileged CHiSP sample. The prestige of the father’s occupation at age 16 (FAPREST) was scored according to Treiman (1977), with a mean of 48.8 and a standard deviation of 13.5 among 983 valid values.

Grades were standardized within each of the 121 school classes of PS16, averaged for the 4 or 5 most important subjects in each branch of the *Gymnasium* (Meulemann, 1979, p. 195), and adjusted so that the highest values represented the highest achievement. The mean of AVGRADE was 2.03, and the standard deviation was 7.04 for 997 respondents. As for aspirations, plans for the *Abitur* and for further study were surveyed and combined (Meulemann, 1979, pp. 72–5). Of 999 respondents, (1) 40.4% definitely aspired to the *Abitur* and study, (2) 14.4% aimed for the *Abitur* and probably study, (3) 20.1% aimed for the *Abitur* but were undecided about study, and (4) 23.7% were heading for occupational training or were undecided. A dummy variable ABISTUD was constructed for the first category, a dummy variable ABIOPEN for the second and third categories, while the fourth served as the base with a value of 0. Finally, students were asked whether they had “an idea of what you want to achieve sometime in life” (Meulemann, 1979, p. 141). Of 999 respondents, 20.3% had “a definite life goal,” 58.5% had “general ideas,” and 19.7% had “no precise ideas.” A dummy variable LIFEGOAL was constructed for the first category, with the other two as the base with a value of 0.

### 3.4 Testing the hypotheses

The *effectiveness* (H1) and the *control force* (H2) hypotheses are tested in the *career take-off from age 16 to 30* using OLS regressions. Starting conditions that do not confirm the effectiveness hypotheses during the career take-off are no longer considered in the analysis of the *life-long career from 16 to 66* using HLM 8 (Raudenbush et al., 2019). For the remaining effects, HLM tests (H1) and (H2) again, and additionally the *advancement* (H3), *tapering off* (H4), and *control persistence* (H5) hypotheses. It follows three steps.

First, the relative weight of the two analysis levels—development during life and persistent starting conditions—must be assessed. M1, the ANOVA model without predictors, serves this purpose. It estimates the general mean of the dependent variable and the between- and within-subject variances required for the computation of the Intraclass Correlation Coefficient (ICC). The ICC is defined as the quotient of the between-subject variance over the sum of the between- and within-subject variance. In our case, the between-subject variance is predicted by starting conditions, while the within-subject variance reflects their development from age 30 to age 66.

Second, the form of development on level 1—within each subject—must be ascertained. M2 and M3 serve this purpose. M2 estimates an intercept and tests the linear slope of age. M3 additionally tests a quadratic effect of age. To minimize the correlation between age and age squared, ages 30, 43, 55, and 66 were recoded as deviations from the approximate mean of 48 into a variable AGE48 with values −18, −5, 8, and 18; however, the original numbers are retained in the presentation for reference to the panel waves. If M3 performs better than M2, linear slopes and quadratic effects of age are introduced in models M4 and M5; if not, only linear slopes will be retained. The *advancement hypothesis* (H3) is confirmed by a positive linear and negative quadratic coefficient for age—that is, by a decelerating increase—in M3.

Third, the effects of the starting conditions on level 2, given the development on level 1, must be examined. M4 (main effect model) and M5 (cross-level interaction model) serve this purpose. In both models, the effects of age re-appear according to the superior models M2 and M3, making their absorption by the effects of starting conditions visible. M4 examines the main effects of the starting conditions on prestige and income. So far, it is comparable to the OLS regressions on career take-off, presented in Table 2 below; yet, in contrast to the OLS regression, it contains coefficients for age. The *effectiveness hypothesis* (H1) is confirmed by significant coefficients of the starting conditions, while the *control force hypothesis* (H2) is supported by smaller *standardized* coefficients for given than for controllable starting conditions in M4.

**Table 2:** OLS regression of MPS and HINC at age 30 on starting conditions at age 16.

	MPS		HINC	
	B (SE)	Beta	B (SE)	Beta
Constant	14.724 (10.185)		13.537 (2.455)**	
MALE	6.768 (2.053)**	0.105	0.462 (0.497)	0.034
IST	0.202 (0.094)*	0.070	−0.041 (0.023)	−0.067
FAPREST	0.472 (0.077)**	0.196	0.024 (0.018)	0.048
AVGRADE	9.688 (1.498)**	0.213	0.338 (0.360)	0.035
ABIOPEN	14.940 (2.662)**	0.221	1.693 (0.639)**	0.117
ABISTUD	15.939 (2.716)**	0.242	1.340 (0.651)*	0.095
LIFEGOAL	2.133 (2.519)	0.026	−0.247 (0.609)	−0.014
R^2^ (*n*)	0.204 (860)		0.017 (828)	

**p ≤ 0.01, *p ≤ 0.05.

M5 adds starting conditions as predictors of the coefficients for linear and, if confirmed empirically, quadratic age, that is, cross-level interaction effects. It tests the research question of whether the effect of starting conditions at age 30 continues to affect prestige and income up to age 66. If the cross-level interactions between a given starting condition and its linear slope are significantly *positive*, its effect at age 16 on prestige and income re-surfaces even at age 43 and later; if additionally, some quadratic effect appears, it must be added to the linear slope; if only a quadratic effect appears, the re-surfacing is visible at its peak. If the cross-level interactions between a given starting condition and its linear and quadratic slopes are insignificant, their effects on the impact of age 16 on prestige and income remain unaffected by respondent characteristics even at later ages. If the cross-level interactions between a given starting condition and its linear slope are significantly *negative*, its effect at age 16 diminishes in later life, confirming the *tapering off hypothesis* (H4): Time heals wounds. Just as with the re-surfacing, quadratic effects might modify the tapering off. If the negative cross-level interactions are smaller for given than for controllable starting conditions, the *control persistence hypothesis* (H5) is confirmed.

M1 to M5 are hierarchical. Whether a higher-order model performs better than the next lower one, in other words, whether the newly introduced variables as a group *together* improve the model significantly, is tested by the difference in their deviances. In our case, thus, only M4 and M3, M5 and M4 need to be compared. For example, if the difference between M4 and M5 is significant, starting conditions as a group have differential effects on prestige and income for different ages; if it is not, they do not change their impact after age 30.

In each model, the variance–covariance matrix of the independent variables on level 1, that is, ages, must be specified in order to determine the number of parameters of the model. If it is specified as “unrestricted,” parameters for the variances of each age and for the covariances of each age pair are estimated—for 4 ages, 4 variances and 6 covariances, which sum up to 10 estimated parameters. If it is specified as “homogeneous at level 1,” the intercept error variance (sigma square) and a constant covariance (tau) are estimated, and each variance is the sum of both terms, with each covariance only representing the latter, such that only 2 parameters are estimated. The unrestricted model can be tested against the homogeneous one by a likelihood-ratio test. As long as it is significant, there is still something to explain in the data.

To estimate the variance–covariance matrix of prestige and income over the four waves in HLM, their correlations must be sufficiently high. For both dependent variables, the correlations decrease with the distance between ages and increase with their order. However, the correlations for income are consistently lower than those for prestige.

## 4 Results

### 4.1 Career take-off: prestige and income

The *effectiveness* (H1) and the *control force hypotheses* (H2) are examined by the standardized coefficients of OLS regressions of MPS and HINC at age 30 on starting conditions at age 16 in Table 2.

For MPS, the educationally defined aspirations—ABIOPEN and ABISTUD—are the strongest predictors, while the self-defined aspiration LIFEGOAL has no impact at all, and IST has a rather small effect. Thus, the *effectiveness* (H1) and the *control force hypotheses* (H2) are mostly confirmed. For the analysis of the life-long career, LIFEGOAL and IST are dropped, while MALE, FAPREST, AVGRADE, ABIOPEN, and ABISTUD are retained.

The standardized regression coefficients of MPS can be compared with two non-selective American samples following the “Wisconsin model” over approximately the same age range from 15 to 30, which, because the American school system filters students after age 15, are not socially selective (Jencks et al., 1983, pp. 330, 334–5). With some divergence between the predictor sets, the German standardized regression coefficients align with the American ones only in one respect: the effect of intelligence is similarly small. Yet the effects of father’s occupational prestige^8^ and grades are larger in Germany, and the effect of educational aspirations in Germany is greater than the effect of occupational aspirations in the USA. The early social filtering in the German school system suggests the opposite expectation: having already operated as a filter, social background loses its power later on. Instead, the antecedent social selection may have—as stated in the introduction—triggered competition and, consequently, mobilization of background resources.

For HINC, only the educationally defined aspirations are significant and strong predictors. Therefore, only these should be retained for the analysis of the life-long career. Thus, the *effectiveness* (H1) and the *control force hypotheses* (H2) are confirmed only in part. However, to analyze both dependent variables in the same form, MALE, FAPREST, AVGRADE, ABIOPEN, and ABISTUD are also retained.

The coefficients for income can also be compared with the two non-selective samples of the “Wisconsin model” (Jencks et al., 1983, pp. 334–5). In both countries, intelligence and father’s occupational prestige have no significant positive effect on income; intelligence even has a negative effect, significant in one American sample. Intelligence may be associated with socially unwelcome behaviors such as egocentrism and arrogance, which are not yet detectable at the entry into an occupation but become painfully noticeable by peers and bosses deciding on salary promotion. In both countries, grades have no effect—possibly due to similar mechanisms as in the case of intelligence. The effect of educational aspirations in Germany is as small as the effect of occupational aspirations in the USA. As income results at least partly from career performance, effects of starting conditions diminish over time.

In summary, there is more agreement between countries for prestige than for income. Although the social filtering in the American school system occurs later than in the German one, prestige and income follow different developmental patterns in both countries—attainment and conservation versus permanent performance. The difference between the two dependent variables becomes apparent in the R^2^ values: in both countries, occupational prestige can be much better explained than income.

### 4.2 Life-long career: prestige

The hierarchical linear regression models of MPS at ages 30, 43, 56, and 66 on starting conditions at age 16 are presented in Table 3.

**Table 3:** Hierarchical linear models of MPS at ages 30, 43, 56, and 66.

	M1		M2		M3		M4		M5	
Level 1: R^2^ (age: *n* = 3,609)			0.94%		1.01%		18.59%		18.64%	
Level 2: R^2^ (starting conditions: *n* = 975)			0.64%		0.71%		21.11%		21.10%	
	b	t	b	t	b	t	b	t	b	t
Intercept Level 1										
Intercept Level 2	100.099	99.726**	99.937	99.966**	100.618	93.638**	83.820	49.777**	83.079	42.965**
Gender (1: MALE)							9.086	5.183**	10.079	5.162**
FAPREST (centered: 29.76, 0, 33.24)							0.473	6.839**	0.459	5.873**
AVGRADE (centered: −2.64, 0, 2.64)							9.618	7.036**	8.970	6.005**
ABIOPEN (Ref.: no Abi)							14.126	6.950**	13.187	5.478**
ABISTUD (Ref.: no Abi)							17.027	7.519**	18.336	7.055**
AGE48			0.171	6.981**	0.176	7.162**	0.171	6.953**	0.139	2.630**
Gender (1: MALE)									0.047	0.935
FAPREST (centered)									−0.000	−0.003
AVGRADE (centered)									−0.045	−1.336
ABIOPEN (Ref.: no Abi)									−0.008	−0.254
ABISTUD (Ref.: no Abi)									0.016	0.234
AGE48 Squared					−0.004	−2.501**	−0.004	−2.346**	−0.001	−0.303
Gender (1: MALE)									−0.003	−1.038
FAPREST (centered)									0.000	0.533
AVGRADE (centered)									0.002	0.825
ABIOPEN (Ref.: no Abi)									0.004	0.890
ABISTUD (Ref.: no Abi)									−0.006	−1.211
Random part (VC: variance components)	VC	χ2	VC	χ2	VC	χ2	VC	χ2	VC	χ2
Intercept	918.842	15742.0**	931.324	24017.1**	1046.809	15235.2**	843.728	12422.0**	842.715	12416.4**
Age48			0.347	2522.7**	0.403	3254.7**	0.401	3242.6**	0.400	3233.1**
Age48 Squared					0.001	1586.6**	0.001	1581.2**	0.001	1566.3**
Residual Level 1	221.911		134.777		95.159		95.486		95.457	
Deviance unrestricted (No. of parameters)	31094.667 (11)		31047.121 (12)		31040.322 (13)		30802.184 (18)		30796.631 (28)	
Deviance homogeneous (No. of parameters)	32442.101 (3)		32006.249 (6)		31709.318 (10)		31475.010 (15)		31464.754 (25)	
LR-Test unrestricted vs. homogeneous: χ2 (d.f.)	1347.434** (8)		959.128** (6)		668.996** (3)		672.826** (3)		668.123** (3)	

**p ≤ 0.01; * *p* ≤ 0.05, b-coefficients tested one-tailed. MPS: minimum 20.00, mean 33.62, maximum 186.80, standard deviation 33.61. Centered predictors: minimum, mean, maximum in brackets. Full maximum likelihood, M1: ICC: 0.8055. Approximate pseudo R^2^ according to the simplified formula of Snijders and Bosker (1994). Since the level-1 variance of M3 is heterogeneous, *t*- and *p*-values of M4 and M5 are based on robust standard errors that correct for heteroscedasticity of level-1 variance (Wooldridge, 2019). When samples are not obtained by simple random sampling on both levels, the approximate pseudo R^2^ might slightly decrease by adding predictors (Hox et al., 2018, pp. 57–64).

In M1, the empty model, the MPS mean is 100.099. The intercept variance—between persons on level 2—is 918.842; the residual variance—within persons on level 1—is 221.911. The ICC—the variance between persons as the percentage of the total variance—is 918.842/(221.911 + 918.842), which equals 0.8055. Four-fifths of the MPS variance originates from differences between persons, while only one-fifth comes from the occupational life course. Prestige is strongly determined by personal qualities that persistently shape biographical development and varies relatively weakly according to unmeasured personal qualities such as health, personality, preferences, luck, and the vicissitudes of the economy such as business cycles, firm closures, and technological innovations.

In M2 and M3, the effect of AGE48 is significantly positive, and in M3, the quadratic effect of age is significantly negative. Prestige increases at a decelerating rate during the career, in accordance with the *advancement hypothesis* (H3). However, as shown in the two top lines, age does not explain much: M2 accounts for 0.94% of the variance at level 1 and 0.64% at level 2; M3 accounts for 1.01 and 0.71%. In both models, slightly more variance is explained at the level where the only predictor, age, operates.

In M4, starting conditions are added to age. The effects of linear and quadratic age remain constant, and all starting conditions have significant effects. Age and starting conditions operate independently throughout the life course. As starting conditions have been selected according to their effectiveness in the analysis of career take-off in Table 2, this is not surprising. Nevertheless, the persistence of age effects reveals something new: Just as countries, age values are “proper names” without informative content (Przeworski and Teune, 1970, p. 8). Just as country names must be interpreted through constant or changing country-level qualities such as religious tradition or GDP, so must age values be interpreted through persistent or malleable personal characteristics—namely, the starting conditions examined here and unmeasured competing qualities such as heredity, health, personality, and preferences. Even in this competition, MALE, FAPREST, AVGRADE, ABIOPEN, and ABISTUD maintain their impact on MPS throughout the life-long occupational career. The explained variances at both levels increase less strongly at level 1 (from 1 to 19%) than at level 2, where the starting conditions operate (from 1 to 21%). The *effectiveness hypothesis* (H1) is also confirmed in the life-long occupational career.

The effects of starting conditions as a group on prestige are tested by the Chi-square deviance difference between M3 and M4 with homogeneous variances: 31709.318–31475.010 = 234.309, with 5 degrees of freedom significant at *p* < 0.01. The *effectiveness hypothesis* (H1) is confirmed in the life-long occupational career for starting conditions as a group as well.

To examine the *control force hypothesis* (H2) among the starting conditions, the standardized coefficients must be computed: 0.135 for MALE and 0.188 for FAPREST; 0.203 for AVGRADE; 0.300 for ABIOPEN; and 0.247 for ABISTUD. Among the starting conditions effective in career take-off, the given conditions have smaller impacts than controllable ones. The *control force hypothesis* (H2) is confirmed.

In M5, the effects of the starting conditions on AGE48 and AGE48 squared—cross-level interactions between age at level 1 and starting conditions at level 2—are introduced. The *conditional* positive linear AGE48 effect decreases but remains significant. Whereas in M4 it amounted to 0.171 for the total group, it shrinks in M5 to 0.139 for women without study aspirations and with mean father’s prestige and mean grade. The effect of AGE48 squared disappears. None of the cross-level interactions is significant. Age and starting conditions work independently of each other; the development of the effects of starting conditions need not be regarded. This also holds for the cross-level interaction effects of starting conditions as a group. The Chi-square deviance difference between M4 and M5 decreases with a value of 10.256, which is not significant for 10 degrees of freedom (*p* = 0.419). The more parsimonious model is the better one. The explained variances do not increase from M4 to M5. In M5, there are no significant negative interaction effects between AGE48 or AGE48 squared, so the *tapering off* (H4) is not confirmed, and the *control persistence hypothesis* (H5) cannot be confirmed.

Summing up the results of M4 and M5: Individually and as a group, starting conditions impact prestige at career take-off, which persists and changes neither for the better nor for the worse. Although the given starting conditions have a weaker impact than controllable ones, the effects of both do not taper off.

As for the *given* starting conditions evaluable from the standpoint of equality, this summary is ambivalent. In a privileged group, early privileges remain fruitful over a lifetime, which can be viewed negatively. However, there is also no refreshing of opportunities, which can be evaluated positively. Once privileging starting conditions have shown their benign effects in an already privileged group, they cannot be mobilized benignly again. Specifically regarding the two given starting conditions, gender and social origin: Once men have attained higher prestige positions in the early occupational career, they are not privileged again in mid-life, for example, by the opportunity to disregard family obligations. Once parents from higher social origins have assigned their offspring to better educational and occupational careers, they cannot successfully interfere in favor of their offspring in the later occupational competition.

As for the *controllable* starting conditions not evaluable from the standpoint of quality, this summary has a positive implication. Even among the privileged, further privileges are not gained solely based on gender and social origin, but also on grades and aspirations. Achievement and aspirations maintain their momentum; possibly, they are rooted in constant personality traits. Against the power of unfortunate circumstances, personal effort can be successfully mobilized.

### 4.3 Life-long career: income

The hierarchical linear models of HINC at ages 30, 43, 56, and 66 based on starting conditions at age 16 are presented in Table 4.

**Table 4:** Hierarchical linear models of HINC at ages 30, 43, 56, and 66.

	M1		M2		M4		M5		M5R	
Level 1: R^2^ (age: *n* = 3,537)			3.20%		4.20%		4.49%		4.41%	
Level 2: R^2^ (starting conditions: *n* = 917)			1.00%		2.69%		2.59%		2.65%	
	b	t	b	t	b	t	b	t	b	t
Intercept Level 1										
Intercept Level 2	17.689	33.653**	17.460	33.351**	14.185	17.770**	14.118	17.748**	14.166	17.685**
Gender (1: MALE)					3.114	3.316**	3.012	3.311**	2.988	3.298**
FAPREST (centered; −29.79, 0, 33.21)					0.033	0.945	0.033	0.970	0.033	0.948
AVGRADE (centered; −2.03, 0, 2.65)					0.898	1.830*	0.857	1.806*	0.893	1.817*
ABIOPEN (Ref.: no Abi)					1.825	1.598	1.896	1.708*	1.871	1.632
ABISTUD (Ref.: no Abi)					2.370	2.218**	2.439	2.329**	2.440	2.269*
AGE48			0.277	13.506**	0.275	9.612**	0.014	2.627**	0.189	8.434**
Gender (1: MALE)							0.150	2.897**	0.159	2.942**
FAPREST (centered)							0.002	0.641		
AVGRADE (centered)							0.040	1.419		
ABIOPEN (Ref.: no Abi)							0.068	0.918		
ABISTUD (Ref.: no Abi)							0.085	1.350		
Random part (VC: variance components)	VC	χ2	VC	χ2	VC	χ2	VC	χ2	VC	χ2
Intercept	190.538	3508.8**	192.928	3723.9**	188.368	3657.5**	189.313	3696.0**	188.929	3682.1**
Age48										
Residual level 1	272.094		254.897		254.842		252.539		253.312	
Deviance unrestricted (No. of parameters)	28893.365 (11)		28789.229 (12)		28775.198 (17)		28762.449 (22)		28765.252 (18)	
Deviance homogeneous (No. of parameters)	31085.015 (3)		30908.296 (4)		30890.885 (9)		30868.652 (14)		30875.896 (10)	
LR-Test unrestricted vs. homogeneous: χ2 (d.f.)	2191.650** (8)		2119.067** (8)		2115.688** (8)		2106.203** (8)		2110.644** (8)	

**p ≤ 0.01; * *p* ≤ 0.05, b-coefficients tested one-tailed. HINC: minimum 0 €, mean 18.26 €, maximum 478.73 €, standard deviation 2,37 €. Centered predictors: minimum, mean, maximum in parentheses; full maximum likelihood, M1: ICC: 0.4118. Since the level-1 variance of M2 is heterogeneous, the reported *t*- and *p*-values of M3 to M5 are based on robust standard errors. Approximate pseudo R^2^ according to the simplified formula of Snijders and Bosker (1994). When samples are not obtained by simple random sampling on both levels, the approximate pseudo R^2^ might slightly decrease by adding predictors (Hox et al., 2018, pp. 57–64).

In M1, the empty model, the mean income is almost €18. The ICC—the variance between persons as a percentage of the total variance—is 0.4118. Two-fifths of the income variance originates from differences between persons, and three-fifths from the occupational life course. Income is less strongly determined by personal qualities—by the starting conditions examined here and by other unmeasured circumstances.

In M2 and M3, the effect of AGE48 is significantly positive; however, in M3, which is not presented, the effect of AGE48 squared is nil. Income increases consistently by €0.28 per year during the life career. The vicissitudes of the economy follow their own laws rather than developmental patterns of occupational careers. M2 explains 3.20% of the variance at level 1 and 1.00% at level 2—again more when age is a predictor. Only the linear term of the *advancement hypothesis* (H3) is confirmed, and its quadratic term need not be considered in M4 and M5.

In M4, starting conditions are added to AGE48. Three have significant effects at least at *p* < 0.05. Men earn on average €3.11 more than women. With a scale-point increase in AVGRADE, income increases by €0.90. Students aspiring to the *Abitur* and studies at age 16 earn €2.37 more than students without these aspirations from age 30 onwards, and students aspiring only to the *Abitur* (non-significantly) earn €1.83 more. The *effectiveness hypothesis* (H1) is confirmed for gender, achievement, and study aspirations.

To examine the *control force hypothesis* (H2), standardized coefficients must be computed: MALE 0.073, AVGRADE 0.030, ABISTUD 0.053. Among the significant starting conditions, the given condition of gender has no smaller effects than the controllable ones of grades and aspirations; thus, the *control force hypothesis* (H2) is not confirmed. Assuming that income is primarily determined by personal exertion, one might have expected the opposite. The advantage of given conditions over controllable ones may be due to their quality: Gender is visible and for most people constant; it even constrains some occupational recruitment fields; grades and aspirations tend to be forgotten in later life. Nevertheless, AVGRADE and ABIOBEN with ABISTUD have a sizable monotonically positive effect, while the given social condition FAPREST has none. Privileges among the privileged are granted based on gender, achievement, and motivation, but not on social origin. M4 explains 4.20% of the variance at level 1 and 2.69% at level 2; in comparison to M2, the increase is stronger at level 2, where the new predictors are introduced.

The deviance decreases from M2 to M4 with homogeneous variances by a Chi-square value of 17.411, which is significant at *p* < 0.01 for five degrees of freedom. Thus, the effects of MALE, AVGRADE, and ABISTUD are strong enough to indicate an effect of starting conditions as a group, making it worthwhile to examine their development.

In M5, the cross-level interactions between AGE48 at level 1 and the five starting conditions at level 2 are added, such that the main effect of AGE48 refers to the conditional effect of a combination of zero values, namely female, mean of FAPREST, mean of AVGRADE, and no aspiration. In the unconditional model M4, the conditional AGE48 effect decreases from €0.28 to €0.01 per year and remains significant. Of the cross-level interactions, only the one for MALE is significant. If FAPREST, AVGRADE, ABIOPEN, and ABISTUD are zero and interact with AGE48, HINC increases for women by €0.01 and for men by €0.01 + €0.15 = €0.16 per year. Men have not only an advantage in income but also in income increase. Seemingly, most—but not all—of the AGE48 effect in M4 reappears in the cross-level interaction of AGE48 and MALE; most of the income increase occurs among men. In comparison to M4, the explained variance does not increase at either level. Because in M5 MALE, AVGRADE, ABIOPEN, and ABISTUD have positive cross-level interaction effects rather than the expected negative ones, the *tapering off* (H4) is not confirmed; and because only MALE interacts significantly with AGE48, the *control persistence hypothesis* (H5) is also not confirmed.

The homogeneous deviance decreases from M4 to M5 by a Chi-square value of 22.234, which is significant at p < 0.01 for five degrees of freedom. Thus, the strong reduction of the age effect and the two significant gender effects suggest eliminating the non-significant cross-level effects of the starting conditions.

In M5R, the cross-level interactions are reduced to just one with MALE, so the main effect of AGE48 remains a conditional effect, this time restricted to females only while holding the other predictors constant. Compared to M4, the AGE48 effect is still reduced, but less dramatically, from €0.28 to €0.19. In comparison to M5, the cross-level interaction of AGE48 and MALE increases further: If FAPREST, AVGRADE, ABIOPEN, and ABISTUD do *not* interact with AGE48, the yearly HINC increases for women by €0.19 and for men by €0.19 + €0.16 = €0.35, meaning the male advantage in income increase remains nearly the same. The deviance increases from M5 to M5R by a Chi-square value of 7.244, which is insignificant for 4 degrees of freedom (*p* = 0.122); omitting the interactions of the other starting conditions does not significantly compromise explanatory power. Moreover, the explained variances are nearly the same in M5 and M5R.

The strong increase of the AGE48 effect from M5 to M5R raises the question of what it conceals. It cannot be attributed to unmeasured concomitants of age, as they were controlled for in both models. Rather, it suggests that the insignificant interaction effects of age and starting conditions in M5, which are positive, have merged into the age effect for females in M5R. Their effects may have been compelled to re-surface as an increase in the age effect from M5 to M5R.

The strong gender–age interaction in M5 and M5R raises the question of when the gender gap widens after age 30. To find out, four regressions of HINC at ages 30, 43, 56, and 66 were computed, analyzing all starting conditions from Table 1 and additionally considering earlier occupational career success—the attainment of the *Abitur* at age 30, and HINC in the respective preceding wave at ages 30, 43, and 66. Thus, the phase-specific impact of gender can be detected. Men had an advantage of 0.43, 2.61, 1.63, and 1.98 at ages 30, 43, 56, and 66, with the advantage of 2.61 at age 43 being significant at *p* < 0.05. Because in a group of former *Gymnasium* students, job entry and family formation occur later than in the general population, women feel the pressure of family responsibilities and the necessity to downplay career concerns later as well—not yet at age 30, but at age 43, and no longer at ages 56 and 66. The income gender gap opens at age 43 and remains constant thereafter.

In summary, gender, grades, and study aspirations impact income. Men have advantages in both income and income growth. Grades and study aspirations are reflected in income, but neither less nor more so over time. From the perspective of the reproduction of social inequality, this is ambivalent: On the one hand, the given condition of gender, because of its prominence, remains decisive throughout the life course; on the other hand, the controllable conditions of achievement and motivation become productive for attaining income, at least during the career take-off.

### 4.4 Summary and comparisons

This study investigated privileges among the privileged. It began with German high school students in the 10^th^ grade, who had already been selected based on the criteria of gender, intelligence, social origin, and controllable factors of achievement and motivation. It aimed to determine whether the starting conditions at age 16 still affect occupational success—prestige and income—at ages 30, 43, 56, and 66. On the one hand, the pre-selection has already exhausted much of the power of the criteria on which it was based. On the other hand, it may have intensified competition among the privileged and created new privileges. If the pre-selection followed controllable starting conditions, particularly achievement, the starting conditions should show no effects on later occupational success. If the pre-selection did not do so or created new privileges, starting conditions should still exert their power over later occupational success.

The *career take-off* from age 16 to 30 was examined using OLS regressions of prestige and income at age 30 based on starting conditions at age 16. The results differed significantly between the two dimensions of occupational success: The *effectiveness* (H1) and the *control force hypotheses* (H2) were sufficiently confirmed for prestige only. Moreover, prestige was much better explained by starting conditions than income. Finally, intelligence and life goals did not have the expected positive effects on either measure, leading to their exclusion from the predictor set for the life-long career.

The *life-long career* from ages 16 to 30, 43, 56, and 66 was analyzed using hierarchical linear models of occupational success based on age and starting conditions at levels 1 and 2. The results again differed significantly between the two dimensions of occupational success. For MPS and HINC, Table 5 presents the common hypotheses and models and contrasts the differences in regression coefficients and global regression measures—the ICC, the impact of starting conditions relative to development; and the R^2^ values for levels 1 and 2, i.e., age and starting conditions.

**Table 5:** Hypotheses on starting conditions, significant regression coefficients, and global measures in the relevant hierarchical linear models of prestige and hourly net income.

Hypothesis	Model	MPS	HINC
Effectiveness (H1)	M4	+ all starting conditions	+ MALE, AVGRADE, ABISTUD − FAPREST, ABIOPEN
Control force (H2)	M4	+ MALE, FAPREST, AVGRADE, ABIOPEN, ABISTUD	-
Advancement (H3)	M2	+ AGE48	+ AGE48
	M3	+ AGE48 square	− AGE48 square
Tapering off (H4)	M5	-	− positive effects of MALE
Control persistence (H5)	M5	-	− increase of positive effect of MALE
ICC	M1	0.8055	0.4118
R^2^ Age	M2	0.94%	3.20%
R^2^ Starting conditions	M2	0.64%	1.00%

+ Confirmation, − disconfirmation of hypothesis for the listed variables.

Regarding the regression coefficients of MPS, all starting conditions had significantly positive effects in M4, as expected, and they were somewhat lower for given than for controllable ones, also as anticipated. Thus, the *effectiveness* (H1) and the *control force* (H2) hypotheses were confirmed. Furthermore, AGE48 had a significant positive effect, while AGE48 squared had the expected negative effect—together indicating a decelerating increase. Thus, the *advancement hypothesis* (H3) was confirmed. Finally, all starting condition effects persisted with equal strength. Thus, the *tapering off hypothesis* (H4) and the *control persistence hypothesis* (H5) were not confirmed.

For the regression coefficients of HINC, only gender and, to a lesser extent, grades and aspirations had significantly expected positive effects in M4. Thus, neither the *effectiveness* (H1) nor the *control force* (H2) hypotheses were confirmed. Furthermore, age had a positive linear effect and no quadratic effect—together indicating a constant increase. Thus, the *advancement hypothesis* (H3) was confirmed only partly. Finally, only the effect of gender increased. Thus, the *tapering off hypothesis* (H4) and the *control persistence hypotheses* (H5) were not confirmed.

The assumption that given starting conditions have weaker and less persistent effects than controllable ones found only limited support. The *control force hypothesis* (H2) is confirmed only for MPS, while the *control persistence hypothesis* (H5) is not confirmed for either of the two dependent variables. Disappointingly from an agency standpoint, success in privileged careers is more strongly a matter of inherited capital rather than personal endeavor. Endowment matters more than attainment, nature more than merit.

When comparing the regression coefficients between MPS and HINC, starting conditions have a strong and persistent impact on MPS, while only gender, grades, and aspirations affect HINC. Furthermore, MPS is negatively impacted by AGE48 squared, in addition to the small positive linear AGE48 effect. An inverted U shape is added to an upward trend—visualizable as the upper curve of a drawbridge raised halfway. Students attain prestige early and gain less over time. The developmental law of prestige is: early attainment for the sake of safe lifelong returns. Income, however, does not depend on age squared, but only on linear age. It increases continuously without deceleration or acceleration. Its developmental law is permanent improvement.

For the global measures of MPS, the ICC in M1 shows that four-fifths of the variance is attributed to starting conditions rather than development; and the R^2^ values in M2 indicate that age explains only slightly more variance than starting conditions. For the global measures of HINC, the ICC shows that two-fifths of the variance is attributed to starting conditions rather than development; and the R^2^ indicates that age explains almost three times as much variance as starting conditions. MPS is determined less strongly by age than by starting conditions, while for HINC, it is the opposite.

In sum, prestige and income follow different developmental forms, as expected in the conceptual discussion in section 3.1. Prestige is attained early according to background qualities and consolidated later; income evolves continuously according to performances that are permanently re-assessed. The difference in developmental forms suggests a difference in the functions of the two status dimensions over the life course, which are often viewed as equivalent from a cross-sectional perspective. Prestige reflects starting conditions and may serve as a means to maintain inherited advantages. A teacher’s child who becomes a teacher can relatively early succeed in preserving the family tradition. Income is largely independent of starting positions; it may serve as a pathway to compensate for earlier disadvantages. A worker’s child who attends university, even without graduating, may, through hard work later on, advance to well-paid occupational positions.

## 5 Conclusion: strengths and limitations

The strength of the study is that it follows a cohort throughout their entire occupational career—from ages 16, 30, 43, 56 to age 66, and from the years 1969, 1984, 1997, 2010 to 2020. Regularly, the cohort should have attained the *Abitur* by 1973 and a university graduation by 1978, that is, before the second panel wave in 1984. However, the study’s focus on a single cohort *only* is also its first limitation: It cannot disentangle the effects of biographical factors from those of historical time.

However, historical events can trigger effects on occupational careers only if their specific biographical relevance is identified. Even if changes in historical time are detected, it must be clarified how they affect the studied cohort. For example, in this cohort, males had advantages in prestige at age 30 in 1984 (see Table 3). However, the percentage of students with access to higher education in Germany increased from 20.4% in 1975 and 28.3% in 1984 to 36.9% (https://www.datenportal.bmbf.de/portal/de/grafik-2.5.85.html. 05.01.2025) such that both genders to attain higher prestige positions and reducing the male advantage. Indeed, women with access to higher education surpassed men between 2000 and 2020; women advanced from 33.5 to 62.2%, while men improved from 33.2% to only 51.4% (BMBF, 2023, p. 44). However, women could not maintain this advantage through to university graduation (BMBF, 2023, p. 55). Thus, the conditions that caused a male advantage in 1984 disappeared by 1997 and resurfaced by 2020; advantages gained in later cohorts were lost again during their careers.

More generally, *Gymnasium* attendance has increased significantly from 19 and 23% in 1965 and 1970—the attendance time span for this cohort—to a stable 38% from 2010 to 2020 (BMBF, 2023, p. 34). However, this educational expansion has not only enlarged the privileged population but also intensified competition within it, suggesting that our results may have gained rather than lost relevance. This may particularly explain the surprising finding that given starting conditions have a stronger impact on occupational careers than controllable ones. The intensified competition in an expanding selective secondary school system seems to strengthen the impact of given rather than controllable starting conditions.

A second limitation of the study is that the time intervals between waves are fairly long. However, longer time spans may increase rather than decrease effects because employees need time to learn about their capabilities. Dunifon and Duncan (1998, p. 43, 46), showed that shorter time intervals in earlier studies and between earlier and later analyses within the same study had weaker effects of motivational factors on occupational success.

A third limitation is that the starting conditions were not sufficiently surveyed in the first survey. In particular, life plans at age 16 were surveyed only superficially—regarding their existence but not their content—resulting in an important aspect of career motivation being underexplored. If surveyed more thoroughly, controllable starting conditions might have fared better than given ones.

## Data Availability

The raw data supporting the conclusions of this article will be made available by the authors, without undue reservation.

## Footnotes

^1^Both figures have changed dramatically. In 2020, 38% of the 8^th^ grade students attended the *Gymnasium*. In 2007, 50% of the high school leavers came from the *Gymnasium*.

^2^While there is much research on the determinants of aspiration (references in Hillmert et al., 2023), we found only the few studies above on their consequences in the life course.

^3^Over 50 years the CHiSP lost about 69% of the initial sample. This resulted in a small success bias, which, however, could only be observed for some socially visible variables. The largest attrition was found for the attainment of a university degree: in FU30, 57.5% reached a university degree. This percentage increases significantly to 64.0% in the sample of FU66. The mean value of the last occupational prestige (MPS) measured in FU30 (92.6) increases significantly by 2.7 points up to FU66. No bias was found for income. Just as for the earlier follow-up surveys, the data collection for FU66 was funded by the German Research Foundation (DFG-Az. Bi 683/3–1/2, ME 577/18–1/2).

^4^Magnitude scaling rests on judgements of the ratio of the prestige of different occupations to the prestige of a base occupation given by respondents of a nationally representative sample. It aims to capture the structural, career-guiding aspect of prestige.

^5^We prefer the median to the mean, in order to minimize the weight of extreme responses.

^6^In contrast, agency refers to “beliefs about one’s ability to act successfully” and “perceptions of … current and future capacities” (Hitlin and Johnson, 2015, p. 1430, 1434). It is a matter of realistic self-assessment rather than intention, of expectations rather than aspirations.

^7^The IST is a speed test. Its calibration used four subtests: Word Knowledge, Reasoning by Analogy, Extending Number Series, Representation of three in two Dimensions; the first two are verbal, the last two non-verbal tests.

^8^As the American sample uses father’s education, mother’s education, and family income as additional background predictors, some of the impact of father’s occupational prestige may have been taken over by them. However, none of them has a significant positive effect, and some even a negative one.
